# Complex Inferential Processes Are Needed for Implicature Comprehension, but Not for Implicature Production

**DOI:** 10.3389/fpsyg.2020.556667

**Published:** 2021-01-05

**Authors:** Irene Mognon, Simone A. Sprenger, Sanne J. M. Kuijper, Petra Hendriks

**Affiliations:** ^1^Center for Language and Cognition Groningen, University of Groningen, Groningen, Netherlands; ^2^Department of Inclusive and Special Needs Education, Faculty of Behavioral and Social Sciences, University of Groningen, Groningen, Netherlands

**Keywords:** scalar implicatures, language acquisition, horn scales, asymmetries, semantics–pragmatics interface, optimality theory

## Abstract

Upon hearing “Some of Michelangelo’s sculptures are in Rome,” adults can easily generate a scalar implicature and infer that the intended meaning of the utterance corresponds to “Some but not all Michelangelo’s sculptures are in Rome.” Comprehension experiments show that preschoolers struggle with this kind of inference until at least 5 years of age. Surprisingly, the few studies having investigated children’s production of scalar expressions like *some* and *all* suggest that production is adult-like already in their third year of life. Thus, children’s production of implicatures seems to develop at least 2 years before their comprehension of implicatures. In this paper, we present a novel account of scalar implicature generation in the framework of Bidirectional Optimality Theory: the Asymmetry Account. We show that the production–comprehension asymmetry is predicted to emerge because the comprehension of *some* requires the hearer to consider the speaker’s perspective, but the production of *some* does not require the speaker to consider the hearer’s perspective. Hence, children’s comprehension of scalar expressions, but not their production of scalar expressions, is predicted to be related to their theory of mind development. Not possessing fully developed theory of mind abilities yet, children thus have difficulty in comprehending scalar expressions such as *some* in an adult-like way. Our account also explains why variable performance is found in experimental studies testing children’s ability to generate scalar implicatures; moreover, it describes the differences between children’s and adults’ implicature generation in terms of their ability to recursively apply theory of mind; finally, it sheds new light on the question why the interpretation of numerals does not require implicature generation.

## Introduction

From the earliest age, humans exhibit extraordinary communicative abilities and a pro-social, cooperative attitude. By their first year of life, for instance, infants are able to use nonverbal pointing gestures to direct other individuals’ attention (Carpenter et al., 1998) and, just a few months later, they appear to grasp the cooperative and mental essence of communication: from 18 months of age, infants can interpret pointing gestures on the basis of the experience they have shared with others (Liebal et al., 2009), and tend to repair episodes of miscommunication irrespective of whether the result of the communicative act is in their favor (Grosse et al., 2010). Moreover, some studies demonstrate the existence of a relationship between early pragmatic abilities such as gaze following and pointing and later language development (Brooks and Meltzoff, 2005; Colonnesi et al., 2010), suggesting that the pragmatic component plays a critical role in language acquisition in general. In light of this, children’s difficulties with particular forms of pragmatic inferencing appear rather puzzling. In the last two decades, a steadily growing body of literature has focused in particular on Scalar Implicatures (SIs) (Noveck, 2001; Papafragou and Musolino, 2003; Guasti et al., 2005; Barner et al., 2011; Foppolo et al., 2012; Stiller et al., 2015; Skordos and Papafragou, 2016, among others). Consider the sentence in (1), which adults normally interpret as (2):

(1) Some roses in William’s garden are red.
(2) Some but not all roses in William’s garden are red.

According to the classical Gricean account of SI generation (Horn, 1972; Grice, 1975; Gazdar, 1979), listeners infer (2) from (1) because of the presence of a non-pronounced alternative utterance, namely (3):

(3) All roses in William’s garden are red.

Even though the semantic (literal) meaning of *some* is AT LEAST ONE, POSSIBLY ALL (notice that forms are presented in italics and meanings are presented in small caps), the quantifiers *some* and *all* are considered as being part of a Horn scale, so named after Horn (1972). Horn scales are lexical scales organized by informativeness: *some*, the first element of the scale <*some*, *all*>, is less informative than the second element, *all*. Informativeness is generally considered to be based on the semantic relation of entailment: *all* entails *some*, but not vice versa. When speakers use the less informative term of a Horn scale, uttering sentence (1) instead of sentence (3), they manifestly violate Grice’s Quantity Maxim, according to which cooperative speakers should always provide as much information as possible. To reconcile the apparent violation of the maxim with the expectation that the speaker is cooperative, listeners can infer that the speaker believes that the non-pronounced sentence (3) is not true. Hence, upon hearing the form *some*, and by negating its stronger alternative *all*, the meaning SOME BUT NOT ALL can be pragmatically derived.

It is worth mentioning that different and sometimes conflicting hypotheses concerning SI generation have been proposed. According to the defaultist view developed by Levinson (2000), implicature generation is automatically triggered by the scalar term *some*; so, by default, irrespective of the context, whenever *some* is used, SOME BUT NOT ALL is derived. On the other hand, according to the grammatical view, SIs emerge at the level of semantic computation (Chierchia et al., 2012; see also Magri, 2009, and subsequent works). According to the defaultist approach and the grammatical approach, the SOME BUT NOT ALL meaning of *some* is not considered as emerging from an online pragmatic process, and it should not be referred to as “pragmatic meaning.” In light of this, here we will use the more theory-neutral expression “upper-bounded meaning of *some*” (i.e., the interpretation that excludes the upper bound of the scale, *all*).

Despite knowing the semantic meaning of the quantifier *some* from an early age (Pouscoulous et al., 2007), children struggle to infer its upper-bounded meaning. Until at least 4 or 5 years of age, they tend to accept sentences that for adults would be underinformative, such as sentence (1) in a context in which the full set of roses is in fact red (e.g., Noveck, 2001; Papafragou and Musolino, 2003; Guasti et al., 2005; Pouscoulous et al., 2007; Huang and Snedeker, 2009; Katsos and Bishop, 2011; Foppolo et al., 2012; Skordos and Papafragou, 2016; Horowitz et al., 2018). This issue has been investigated in a large body of literature. However, to date, there is still considerable disagreement about the reason behind children’s non-adult-like behavioral pattern, with some researchers focusing on the detrimental effect of task demand (e.g., Papafragou and Musolino, 2003) and others holding that children’s problems are intrinsically linked to the pragmatic inferencing process (e.g., Huang and Snedeker, 2009).

In this paper, we will propose the Asymmetry Account, a new account of SI generation, couched in the framework of Bidirectional Optimality Theory (Bi-OT). Importantly, Bi-OT allows us to analyze production and comprehension as separate processes (Blutner, 1998, 2000). Moreover, following Hendriks and Spenader (2006) (but *contra* Blutner, 2006, 2010), we will argue that Bi-OT has psychological validity, and we will show that it correctly predicts children’s performance. In particular, we will start presenting some acquisition findings (see section “Different Tasks, Conflicting Results”). We will then introduce two influential accounts of children’s difficulties and illustrate some recent corpus data (Eiteljoerge et al., 2018) that point to the fact that children are able to produce SIs already in their third year of life (see section “Previous Accounts of Children’s Difficulties”). We will see that this finding casts doubts on the idea that children’s difficulties lie in the process of SI generation itself (see section “The Pragmatic Tolerance Account”). We will then show that, contrary to this view, the production–comprehension asymmetry is real (see section “Challenges for the Pragmatic Tolerance Account”). In Section “Carving Quantity-Based Implicature at Its Joints: *Ad Hoc* and Horn Scales,” we will rigorously define and discuss some features of SIs. Then, we will present our Asymmetry Account (see section “The Asymmetry Account: A Cognitively Plausible Model of Children’s Difficulties”) and discuss its predictions (see section “Discussion”). Specifically, we will show that children’s comprehension difficulties emerge because implicature generation imposes a cognitive burden on hearers, but not on speakers. Accordingly, children’s pattern of performance is explained by the fact that complex inferential processes are not needed in production, but only in comprehension (see section “When Speakers Are More Logical Than Hearers”); in Section “Scalar Implicature Generation and Theory of Mind,” the relationship between SIs and theory of mind (ToM) is described; in Section “When Speakers Become Less Logical and More Pragmatic,” the differences between children’s and adults’ ability to generate implicatures are illustrated. This paper ends with a discussion of the reasons behind children’s variable performance in comprehension studies (see section “Explaining Children’s Variable Performance in Comprehension Studies”) and on the reason why children’s interpretation of numerals does not require implicature generation (see section “Why Children Interpret *n* as EXACTLY *n*”).

## Scalar Implicatures in Acquisition

### Different Tasks, Conflicting Results

One of the striking characteristics of studies on children’s implicature generation is that the particular task used and the contextual support provided to participants substantially influences the outcome of the experiments, to such an extent that the age at which children have been reported to acquire the adult-like interpretation varies between age 5 and preadolescence.

Noveck (2001) is one of the first studies to systematically investigate SIs in language acquisition (but see also Paris, 1973; Smith, 1980; Chierchia et al., 1998, 2001). In this study, children were asked to evaluate sentences such as “Some giraffes have long necks” uttered in isolation (Statement Evaluation Task). Noveck’s (2001) results indicated that even at the age of 11, children do not reliably reject underinformative sentences containing the quantifier *some*. However, tested with this paradigm, even the adult participants in this study did not draw inferences at a high rate (59% for adults vs. 15% for 11-year-olds). Hence, albeit being useful in revealing a difference between children and adults, the Statement Evaluation Task does not seem the most reliable tool to measure SI generation, given that, as demonstrated in later studies (e.g., Guasti et al., 2005), this paradigm favors the emergence of logical interpretations also in adult participants. Probably, the reason for this lies in the abstract nature of the task, which consists in judging world-knowledge statements in isolation.

Subsequent studies (Lidz and Musolino, 2002; Papafragou and Musolino, 2003; Guasti et al., 2005; Foppolo et al., 2012) adopted another kind of comprehension task, namely, the binary Truth Value Judgment Task (TVJT), and showed that the age at which children are able to generate SIs can be lowered considerably. For instance, in Experiment 1 of Foppolo et al. (2012), adults and children aged 4 to 7 were asked to evaluate a sentence in combination with a particular picture (e.g., “Some Smurfs are going on a boat” presented in combination with a picture in which five out of five Smurfs are on a boat). Six-year-old children demonstrated to be able to generate SIs almost at an adult-like rate (83% for 6-year-olds vs. 87% for adults).

Interestingly, the same study also illustrates the largely overlooked difference between the ability to generate SIs and the ability to identify the most informative between two given alternatives. In Experiment 5 of Foppolo et al. (2012), the group of 5-year-olds who failed to compute the SI in the previously administered TVJT was administered a Felicity Judgment Task (FJT). In FJTs (a paradigm first introduced by Chierchia et al., 2001), participants are provided with two statements and are asked which one best describes a given picture. In the critical items of Foppolo et al.’s (2012) Experiment 5, children heard a sentence containing *all* and a sentence containing *some* (e.g., “All the chipmunks are taking a shower” vs. “Some chipmunks are taking a shower” in combination with a picture showing five out of five chipmunks taking a shower). Quite surprisingly, children’s performance in this task was 95% correct overall (see also Chierchia et al., 2001, for similar results). Thus, children’s difficulties with SI generation do not appear to emerge in connection with an inability to grasp the difference (in terms of informativeness) between *some*- and *all*-sentences (see section “Previous Accounts of Children’s Difficulties” for discussion).

That the experimental manipulation can drastically influence children’s performance in SI experiments was further demonstrated also by a study conducted by Pouscoulous et al. (2007). These authors adopted an Act-Out Task (AOT), a methodology that allows children to indirectly exhibit their ability to generate SIs by performing an action, instead of giving a verbal judgment. According to the authors, task demand is to be held responsible for hampering children’s SI generation in TVJT and similar paradigms. In line with this hypothesis, their results showed that, if the task is simple enough, from the age of 5, children rather robustly generate SIs: in their task, 73% of 5-year-olds (and 88% of adults) demonstrated to have interpreted *some* as SOME BUT NOT ALL.

### Previous Accounts of Children’s Difficulties

Various explanations have been proposed for why children experience difficulties in generating SIs. In what follows, we discuss two of the most influential accounts: the Lexicalist account by Barner et al. (2011) and the Pragmatic Tolerance account by Katsos and Bishop (2011).

#### The Lexicalist Account

According to Barner et al. (2011), children’s problems do not stem from pragmatic immaturity or processing difficulties, but rather lie in a particular step of SI generation, namely, the retrieval of the scale of alternative lexical terms. In fact, accessing the scale and recognizing the existence of an alternative is clearly a prerequisite for generating implicatures. Barner et al. (2011) argued that preschoolers fail in generating the relevant scalar alternative (e.g., *all*) when hearing a scalar item (e.g., *some*) (see also Foppolo et al., 2012, for a similar claim). Notably, this hypothesis can explain why children struggle with Truth Value Judgment Tasks but show adult-like performance in Felicity Judgment Tasks (Foppolo et al., 2012). In the latter case, the strong alternative (the sentence with *all*) is already given in the task, and the task can be carried out simply by recognizing that in critical trials, the *all*-sentence is more appropriate.

However, as we will now see, further experimental evidence (Eiteljoerge et al., 2018) casts doubt on the plausibility of the lexicalist account.

#### The Pragmatic Tolerance Account

Despite the ever-growing number of studies devoted to the topic, researchers have mainly focused on children’s comprehension of SIs and hardly any experiment examined children’s *production*. There are, however, a few notable exceptions.

Production data are presented by Foppolo and Guasti (2005). In this study, an Elicitation Task was used in order to assess whether children can use *some* and *all* in an adult-like manner. With the aim of eliciting sentences containing quantified NPs, Italian children aged 3;7 to 5;8 were presented with stories and asked to describe what had happened to a set or subset of characters. Children’s mistakes were rather infrequent: *all* was used correctly 95% of the time [only in 4 out of 71 utterances children used *tanti* (many) instead of *tutti* (all) to refer to a full set of characters]. On the other hand, to refer to a subset of characters, children produced 53 utterances containing different lexical items that appear equivalent to the English *some* (the exact number of instances of the different items used is not reported in the paper). Foppolo and Guasti’s (2005) conclusion was that children can appropriately use *all* and *some* in production: the former when describing a full set of relevant characters, the latter when describing a subset of the relevant characters. Importantly, children never used *some* underinformatively to refer to a full set of characters.

A discrepancy between the correct use of quantifiers in production and the difficulties in comprehension emerges also in the study of Katsos and Smith (2010). In Experiment 1, children were tested in both comprehension and production. The comprehension part consisted in a classical TVJT, in which children listened to stories and were asked to indicate whether the fictional character Mr. Caveman replied correctly to some questions. In critical trials, Mr. Caveman would say, for instance, that some of the carrots had been picked up when in fact all of them had been picked up. In the production task, on the other hand, children would see a scenario in which a subset of objects was acted upon; this time, however, not Mr. Caveman, but the children themselves were asked to provide an appropriate description of the situation. Performance in the comprehension task confirmed previous findings: children overwhelmingly failed to reject underinformative sentences, thus showing not to have generated the implicature. The same group of children, however, was able to produce informatively appropriate utterances, using the quantifier *all* (or a numeral, or a plural noun phrase such as *the carrots*) instead of the underinformative *some* when describing the so-called ALL-scenario. So, despite accepting underinformative sentences in comprehension, children demonstrated to be fully informative speakers.

In this study, the intriguing asymmetry between children’s adult-like production and children’s non-adult-like comprehension was interpreted as evidence in favor of the Pragmatic Tolerance Hypothesis (Davies and Katsos, 2010; Katsos and Smith, 2010; Katsos and Bishop, 2011). According to this hypothesis, children are pragmatically competent and are aware of the underinformativeness of *some*-sentences in ALL-scenarios. Nevertheless, they do not penalize pragmatic violations as adults do. As a result, in the binary judgment tasks that are typically employed to test SIs, children tend to accept underinformative sentences—which, in fact, are not semantically false. Nevertheless, in particular paradigms (such as Katsos and Smith’s production experiment), they can exhibit their pragmatic abilities. Their non-adult-like behavior is simply due to an overly tolerant pragmatic attitude.

Further evidence that children in production can show adult-like competence is provided by a recent study carried out by Eiteljoerge et al. (2018). These authors conducted a corpus study analyzing the production of sentences containing the quantifier *some*. Spontaneous utterances (*N* = 2883) of five English children aged 2;00 to 5;01 were inspected and categorized according to the likeliness to contain a SI. The classification was based on the linguistic context (i.e., three lines of context before and after each occurrence of *some* were examined) and structural features (e.g., partitive constructions, plural noun phrases, etc.). An implicature was categorized as *Possible* or *Probable* if a quantifiable set could be recognized and the speaker was probably referring to a subset of the quantified set using *some* with the NOT ALL meaning. Among the included utterances, *Implicature Implausible*-sentences (i.e., sentences in which most likely the speaker was not implying NOT ALL) were the majority (70.76%). Nevertheless, in 19.46% of utterances, an implicature was *Possible* or *Plausible* (e.g., “The puzzle is missing some pieces,” while describing a puzzle). Strikingly, even 2-year-old children were able to use *some* in a way that clearly triggers implicature generation: one child, Fraser, did so at 2;03 years of age; all the others did so before or around 3;00 years of age. In light of their data, Eiteljoerge et al. (2018) criticized Barner et al.’s (2011) lexicalist account, claiming that: “If toddlers have not associated *some* with its lexical scale (*many*, *most*, *all*), this should affect their ability to produce, as well as comprehend, implicatures” (Eiteljoerge et al., 2018, p. 14).

Moreover, as Eiteljoerge et al. (2018) observed, the low rate of produced implicatures in a children’s corpus should not come as a surprise. In fact, children’s production was in line with mothers’ usage, as the analysis of mothers’ child-directed speech revealed. Among adults’ sentences, only 16% of the instances of *some* could be analyzed as *Implicature Possible* or *Plausible*. Interestingly, although in the literature it is almost always implicitly assumed that “scalar implicatures arise more often than not when the lexical item *some* is used” (Degen, 2013, p. 164), this assumption, as shown by Degen (2013), is not borne out by corpus studies.

This being said, the finding that children use *some* with its upper-bounded meaning at least 2 or 3 years before they show an adult-like comprehension of the same term suggests that a purely lexicalist account along the lines of Barner et al. (2011), albeit intriguing, cannot be wholly satisfactory. Moreover, as mentioned by Eiteljoerge et al. (2018), their data are in line with an explanation of children’s non-adult-like comprehension pattern in terms of non-linguistic factors, as proposed by Katsos and Bishop (2011).

#### Challenges for the Pragmatic Tolerance Account

The idea that the difficulties in generating SIs in binary comprehension tasks lie in children’s excessive pragmatic tolerance—and not in the generation itself or in particular steps required for the generation—is extremely appealing, in that it dismisses the issue of the production–comprehension asymmetry. However, a careful examination of further data casts some doubts on the explanatory power of the Pragmatic Tolerance hypothesis.

Firstly, on a general level, it can be argued that if children were more pragmatically tolerant than adults, excesses in pragmatic tolerance would emerge in other contexts too. Contrary to this, however, we know that children are endowed with an astonishing pragmatic sensitivity, which appears incompatible with a hypothetical overly tolerant pragmatic attitude. As an example of children’s extraordinary sensitivity to communicative intentions, consider the aforementioned study of Grosse et al. (2010). In this work, the authors showed that infants as young as 18 months of age recognize and tend to repair episodes of miscommunication even if those same episodes accidentally lead to children’s desired outcomes. In this experiment, children were prompted to ask for an object. In critical trials (*Happy Accident Conditions*), an experimenter would pretend to have misunderstood the request but at the same time accidentally provide the child with the desired object, placing it in a target position. Despite having received the requested object, 18-, 24-, and 30-month-olds tried to repair the communication, through gestures, vocalizations, or verbal sentences.

One could argue that if children were excessively tolerant toward pragmatic violations in general, they would ignore communicative failures and welcome the desired outcome without trying to repair. However, this is not the case. Children as young as 18 months of age do not regard communication as a simple tool to manipulate others’ behavior. On the contrary, they are alert and aware of communicative pragmatic deviances. In light of this observation and of evidence coming from numerous other studies that point to children’s extraordinary pragmatic skills (Matthews, 2014, for an overview), it is safe to claim that children are not, generally speaking, more pragmatically tolerant than adults. Consequently, if pragmatic tolerance is the factor responsible for children’s non-adult-like behavior in SI generation, we have to assume that pragmatic tolerance is restricted to violations of underinformativeness only. This, however, seems an unwelcome result given that we would have to postulate a phenomenon-specific pragmatic tolerance.

Secondly, apart from children’s early pragmatic abilities, it seems quite hard to understand why preschoolers’ pragmatic tolerance would emerge just in comprehension, and not in production too. If children simply required sentences to be true and not also pragmatically appropriate, they should also produce, at least some of the times, pragmatically infelicitous sentences using *some* instead of *all*. However, this does not seem to be the case (Foppolo and Guasti, 2005; Katsos and Smith, 2010).

Thirdly, eye-tracking research (although data are still scarce) seems to suggest that 5-year-old children struggle—or at the very least, require significantly more time than adults—at a processing level, to generate *some*-implicatures (Huang and Snedeker, 2009). If problems emerge in SI processing, the locus of children’s difficulties with SI in general should lie in the inferencing process, or in particular steps of this process. This would be at odds with the Pragmatic Tolerance Hypothesis, according to which there are no inherent difficulties in children’s SI generation.

In sum, although Katsos and Bishop’s (2011) account elegantly explains the asymmetry between the production and comprehension of *some*, it faces substantial challenges and the search for alternative explanations seems to be warranted.

In what follows, adopting the framework of Bidirectional Optimality Theory (Bi-OT), we develop a novel account of children’s SI generation and of the production–comprehension asymmetry that emerges in connection with *some*. As shown by Blutner (1998, 2000), Bi-OT is particularly suited to model Gricean pragmatics (see also Schulz and Van Rooij, 2006; Aloni, 2007; Krifka, 2007, 2010, 2011). We start by rigorously defining SIs, in the belief that any account of children’s difficulties makes terminological clarity particularly important (see section “Carving Quantity-Based Implicature at Its Joints: *Ad Hoc* and Horn Scales”). Then, we describe two constraints that determine the semantics of the scale <*some*, *all*> (see section “Translating Horn Scales in Constraints”). We show how these constraints interact (see section “Constraint Interaction: Modeling Speakers’ and Hearers’ Perspectives Separately”) and how implicatures can be modeled (see section “Bidirectional Optimization: Generating the Implicature”). Lastly, we illustrate the predictions of our Asymmetry Account (see section “Discussion”), which, we argue, explains why children experience difficulties comprehending SIs, although they are able to produce *some* with its upper-bounded meaning from a very young age.

## Carving Quantity-Based Implicature at Its Joints: *Ad Hoc* and Horn Scales

As mentioned in the Section “Introduction,” according to the traditional Gricean approach, conversational implicatures can be seen as non-truth-functional meanings emerging in connection with the Principle of Cooperativity. Quantity-based implicatures (QBIs), in particular, are those implicatures that are based on the two submaxims of Quantity (here in the formulation of Matsumoto, 1995, p. 23).

(4) First submaxim of Quantity: Make your contribution as informative (strong) as possible.
Second submaxim of Quantity: Do not make your contribution more than is required in the context of the exchange.

Under the label QBI, we can include SIs as well as at least some instances of *ad hoc* implicatures. The distinction between scalar (or generalized) implicatures and *ad hoc* (or particularized) implicatures, introduced by Grice (1975, 1989), is based on inferences’ inherent degree of (in)dependence from the context. To illustrate, consider the following sentences and the relevant inferences.

(5) a: I ate some of the apples.
b: I ate some but not all of the apples.
(6) a: My friend wears glasses.
b: My friend wears glasses and not a hat.

The inference in (5b), a SI, appears to naturally follow from the sentence in (5a). In contrast, the inference in (6b), an *ad hoc* implicature, seems not to follow automatically from (6a). In (6a), the implicature emerges only if the context is such that *glasses* and *glasses and hat* constitute relevant alternatives. This happens, for instance, when the sentence in (6a) is uttered in a situation in which there is a person who is wearing a hat and glasses, and another person who is wearing just glasses.

The distinction between *ad hoc* implicatures and SIs has been challenged, among others, by advocates of Relevance Theory (Sperber and Wilson, 1986)^1^. Irrespective of whether the distinction is cognitively legitimate, according to the Gricean tradition, both classes of implicatures are generated when a speaker intends to communicate a particular meaning that goes beyond the literal meaning of the uttered words and does so by uttering a sentence in which the quantity of information is reduced with respect to what the listener could have expected.

Defining formally what is meant with *quantity of information* is rather problematic. A viable solution, proposed by Horn (1972), is to describe *informativeness* in terms of asymmetric semantic entailment. Roughly, an item P asymmetrically entails Q if P is true in all set of circumstances in which Q is true, but not vice versa (see also Gazdar, 1980). To exemplify, if the sentence in (7) is true, the sentence in (8) is also true, but not the other way around.

(7) All of my friends are linguists.
(8) Some of my friends are linguists.

Accordingly, the so-called Horn scales are those ordered sets of lexical items whose members have a similar structural complexity (cf. Katzir, 2007, for an in-depth discussion) and stand in an asymmetrical relationship of entailment, and because of this, are particularly prone to give rise to SIs. So, if in the case of *ad hoc* implicatures, what counts as a relevant alternative is determined by the context (as shown in 6), relevant alternatives are lexically defined in the case of implicatures that emerge from Horn scales (as shown in 5).

It should be observed that, as argued by Hirschberg (1985), the Horn/*ad hoc* scales dichotomy is perhaps a false one. As pointed out by Horn himself in later works (Horn, 2006), what we can call Horn-Scalar Implicatures are, to a certain degree, context-sensitive too. Nevertheless, being based on terms that are strongly associated at the lexical level, they are inevitably *less* context-sensitive than other QBIs (see Barbet and Thierry, 2018, for experimental evidence).

In this regard, it is relevant to mention that the association between the scale mates that constitute Horn scales appears to be demonstrated experimentally. Adopting a masked priming paradigm, de Carvalho et al. (2016) showed that less informative items of scales can prime stronger items of the same scale. Conversely, priming from stronger items to the less informative one is weak. This points to the fact that stronger words are evoked when weaker ones need to be interpreted, but not the other way around. The association operates in one direction only, so scalar weaker terms are *asymmetrically* associated with certain alternatives at the level of the mental lexicon. Scales, in essence, appear to have a psychological reality. Most importantly for our purposes here, the existence of such links between scalar items has been, by and large, taken for granted in the acquisition literature, and the cognitive reality of Horn scales is at the core of Barner et al.’s (2011) lexicalist account.

The controversy in language acquisition is predominantly centered around SIs, strictly defined as being based on the Quantity Maxim and Horn scales. Thus, with the aim of providing an adequate and cognitively plausible explanation of children’s SI generation, as tested in an ever-growing number of studies, we focus on a particular Horn scale, namely, <*some*, *all*>. With slight modifications, the analysis presented in the remainder of this paper, however, can be applied to SIs that emerge from the whole class of Horn scales.

## The Asymmetry Account: A Cognitively Plausible Model of Children’s Difficulties

Describing Scalar Implicature generation presupposes an understanding of Horn scales functioning. Here, we argue that the comprehension and production of *some* and *all*, and consequently, SI generation, are regulated by two semantic principles, or constraints.

Our account is couched in the constraint-based framework of Bidirectional Optimality Theory (Bi-OT). In Bi-OT, production and comprehension of lexical elements are seen as optimization processes in which, given an input, an optimal output needs to be identified. Specifically, in production the input is a meaning and the output is a form (i.e., what will be finally uttered). In comprehension, the input is a form and the output is a meaning (i.e., the interpretation that will be chosen). Clearly, both in production and in comprehension, given an input, there are several possible outputs. When we want to communicate a meaning, we need to choose among different forms, and when we hear a form, we need to choose among different meanings. The evaluation process is guided by constraints. In OT (Hendriks and Spenader, 2006 for OT semantics; cf. Prince and Smolensky, 2004 for OT phonology), these constraints are violable and hierarchically organized. Stronger constraints are more important than weaker ones and, whenever two constraints are in conflict, the weaker one can be violated.

We now show how the interaction between SI constraints can explain children’s comprehension failures as well as their production successes (for a more formal treatment of these constraints and of their interaction in the Bi-OT framework, see Mognon et al., in press).

### Translating Horn Scales in Constraints

The first constraint we introduce arises directly in connection with Grice’s Cooperative Principle. Consider the first submaxim of Quantity, mentioned above in (4). This general and fundamental principle of communication is mirrored in the constraint that we call Strength (cf. Zeevat, 2000, and Hogeweg, 2009, for the production counterpart of this constraint):

(9) Strength: Use the strongest element on the Horn scale.

According to this constraint, if two terms (in this case, *some* and *all*) stand in a relation of entailment and can both be used, *salva veritate*, in a given context, then speakers should lean toward choosing the most informative term (here, *all*).

The constraint Strength interacts with a family of constraints, which, like Strength, is relevant to the comprehension and production of scalar elements. In particular, this family of constraints is essential to introduce a link between forms and the dimension conveyed by Horn scales. First, let us consider a virtually ignored but fundamental feature of scales. Lexical scales are always polarized toward a culmination point, which can be a lower or an upper bound. We call this culmination point the *apex* of the scale. The apex is the maximization of the dimension conveyed by the scale. Equivalently, it represents the strongest lexical meaning of the scale. To give an example, the apex of the scale <*possible*, *certain*> is NECESSITY. It is possible to identify apices also in the case of *ad hoc* scales, even if, needless to say, these are *ad hoc* apices.

To better grasp the nature of apices, consider the following context. A traveler is going from Europe to Vladivostok via the Trans-Siberian route and utters the following:

(10) I’ve reached Novosibirsk.

The utterance in (10) is likely to give rise to a “not Vladivostok”-inference. The *ad hoc* scale here consists of the various stops along the Trans-Siberian route, and the *ad hoc* apex is something like LAST STOP OF TRANS-SIBERIAN ROUTE, which corresponds to the city name *Vladivostok*. It is worth noting that experimentally demonstrating the cognitive reality of scales amounts to demonstrating the cognitive existence of apices. At least for what concerns Horn scales, as mentioned, evidence has already been found (de Carvalho et al., 2016).

Turning back to Horn scales, and in light of the existence of apices, we can now introduce the aforementioned family of constraints: FaithHorn. The family of FaithHorn constraints promotes the mapping between the strongest lexical element on a Horn scale (i.e., the element of the scale that entails the other weaker elements) and a particular meaning, namely, the apex of the relevant Horn scale. When applied to the scale <*warm*, *hot*>, for instance, FaithHorn promotes the mapping between *hot* and the apex of the scale, namely, HEAT. In the case of the <*some*, *all*>-scale, FaithHorn links the term *all* with complete sets. We label this specific constraint FaithAll.

(11) FaithAll: *All* corresponds to complete sets.

Trivial as it seems, FaithAll is a fundamental, primitive constraint of the semantics of the <*some*, *all*>-scale. It is violated by an association between *all* and a non-complete set.

### Constraint Interaction: Modeling Speakers’ and Hearers’ Perspectives Separately

Having introduced the two constraints that are relevant for our account of SIs, we now illustrate their interaction. As mentioned above, in Bi-OT, constraints are seen as violable and hierarchically organized. Complying with a stronger constraint is more important than complying with a weaker constraint, and if two constraints are in conflict, then the weaker constraint can be violated in order to satisfy the stronger constraint.

Production and comprehension of linguistic expressions can be seen as independent but related processes. They are guided by the same constraints, but in production, speakers need to map meanings onto forms, whereas in comprehension, hearers need to map forms onto meanings (Hendriks, 2016). Thus, the effects of the application of the same constraints may yield different results in production and comprehension (Smolensky, 1996).

**FIGURE 1:**
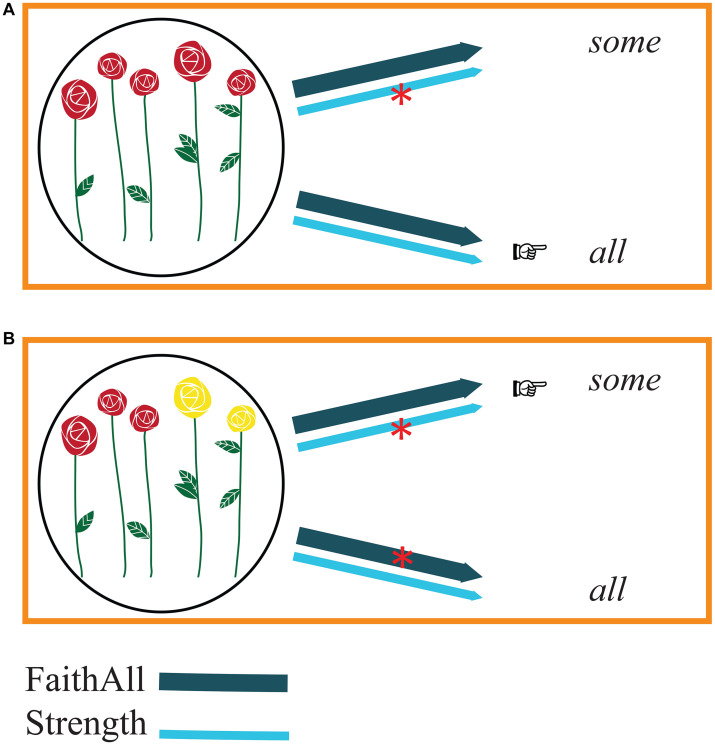
Production (speakers’ perspective). The arrows representing FaithAll (dark blue) and Strength (light blue) link meanings with possible forms. Constraint violations are represented by red asterisks on the arrows. The relative strength of the constraints is indicated by the weight of the line of the arrows: FaithAll is stronger than Strength. Pointing fingers indicate which form proves to be optimal on the basis of the constraints. Panel **(A)** illustrates reference to a complete set of flowers: the meaning to be expressed corresponds to a complete set of elements. In this case, choosing *some* would violate Strength. The optimal form, hence, is *all*. Panel **(B)** illustrates reference to a subset of red flowers in a larger set of flowers of different colors: the situation in which the meaning to be expressed corresponds to a non-complete set of elements. In this case, choosing *some* would violate Strength, whereas choosing *all* would violate FaithAll. However, given that FaithAll is stronger than Strength, the optimal form is *some*.

Let us describe the interaction of constraints, first, taking the perspective of speakers and hence considering the production processes (Figure 1).

Consider a speaker who wants to refer to a complete set of items, in which five out of the five roses are red (Figure 1A). Given the choice between the form *some* and the form *all*, the speaker can easily exclude *some* because choosing it would violate the constraint Strength (“Use the strongest element on the Horn scale”) and generate an underinformative message. Choosing *all* to refer to a complete set, on the other hand, does not violate any constraint: as Strength requires, *all* is the strongest term of the scale at hand and, as stated by FaithAll, can be associated with complete sets. In other words, *all* is the optimal candidate to refer to complete sets. The speaker, thus, can readily utter the following sentence:

(12) All the roses are red.

A different situation arises when the speaker wants to refer to a set that is not complete, where, for instance, three out of the five roses are red (Figure 1B). Choosing *all* violates FaithAll, given that, according to this constraint, *all* should always be associated with a complete set. Choosing *some*, on the other hand, violates Strength, given that there is a stronger term on the scale. However, FaithAll is higher-ranked than Strength. Therefore, the violation of Strength is less grave than the violation of FaithAll. Hence, *some* is a better option than *all* to describe a set that is not complete. So, when a speaker wants to describe a scenario in which three out of five roses are red, using the quantity scale at hand, the speaker’s only option is to utter (13):

(13) Some of the roses are red.

The two production processes just described are carried out by speakers whenever they need to refer to sets using the quantity scale <*some*, *all*>.

**FIGURE 2:**
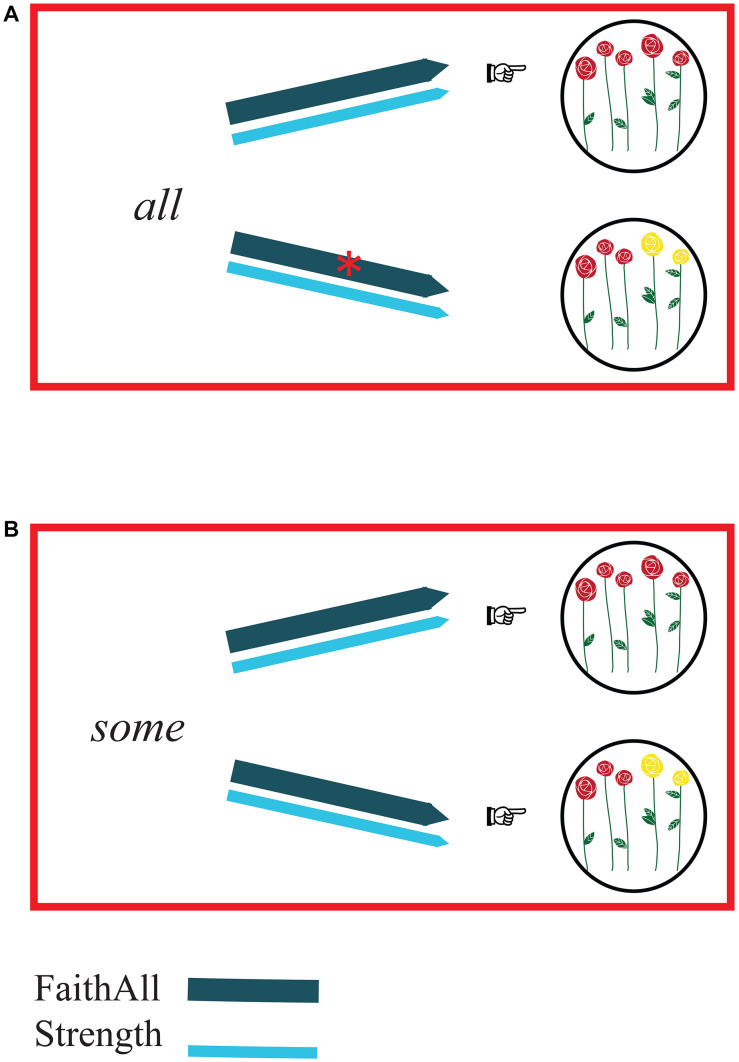
Comprehension (hearers’ perspective). The arrows representing FaithAll (dark blue) and Strength (light blue) link forms with possible meanings. As in Figure 1, constraint violations are represented by red asterisks on the arrows, the relative strength of the constraints is indicated by the weight of the line of the arrows, and pointing fingers indicate which meaning proves to be optimal on the basis of the constraints. Panel **(A)** illustrates the comprehension of the form *all*: FaithAll rules out the interpretation consisting of a non-complete set of elements, whereas Strength does not have any effect. The optimal interpretation appears to be the one consisting of a complete set of elements (here: the complete set of flowers). Panel **(B)** illustrates the comprehension of the form *some*. In this case, both interpretations are possible, because, irrespective of the chosen interpretation, neither FaithAll nor Strength are violated. So, the form *some* turns out to be ambiguous between two interpretations: a complete set of elements (here: the complete set of flowers) and a non-complete set of elements (here: a subset of flowers) are both optimal meanings.

The hearers’ perspective (Figure 2) differs from the speakers’ perspective. In the hearers’ perspective, the constraint Strength has no effect because this constraint expresses a preference for the choice of forms. Hence, it influences production but cannot influence comprehension. In other words, in the comprehension process, the form is already given—it has been uttered by the speaker. Thus, “Prefer the strongest element on the Horn scale” has no effect and it is simply not relevant when deciding how to interpret a form such as *all* or *some*. The comprehension of the elements of the <*some*, *all*>-scale depends uniquely on the constraint FaithAll. How, then, does this constraint affect the interpretation of the two quantifiers?

When the form *all* is heard and needs to be interpreted (Figure 2A), FaithAll (“*All* corresponds to complete sets”) rules out every interpretation but the complete set. Thus, following FaithAll, the form *all* is straightforwardly associated with a complete set meaning.

What about the interpretation of *some*? When the form *some* is heard and needs to be interpreted (Figure 2B), FaithAll does not rule out non-complete sets, nor complete sets: in fact, FaithAll only requires an association between *all* and a complete set. Hence, when *some* has to be interpreted, FaithAll is simply not relevant. So, as outputs of the comprehension process of *some*, complete sets and non-complete sets are both optimal candidates. The result of this is that, from hearers’ perspective, *some* is ambiguous because it is compatible with complete sets and non-complete sets.

The analysis of production and comprehension processes of the <*some*, *all*>-scale just proposed, then, gives rise to the following result: in production, reference to complete sets is made using the form *all* (Figure 1A) and reference to non-complete sets is made using the form *some* (Figure 1B). In comprehension, *all* is straightforwardly interpreted as referring to complete sets (Figure 2A). The comprehension of *some*, on the other hand, is problematic because *some*, in hearers’ perspective, proves to be ambiguous (Figure 2B).

This indeed is what we find when we test children on the comprehension and the production of the most popular Horn scale, <*some*, *all*>: the comprehension and production of *all* are adult-like, and so is the production of the upper-bounded *some* (i.e., SOME BUT NOT ALL). The comprehension of the form *some*, however, is problematic for children: they tend to overaccept *some-*sentences, showing that they do not spontaneously generate the *some*-implicature. This, again, is in line with our model, which predicts that *some* is ambiguous between two interpretations. How do adults resolve this ambiguity that stems from the semantics of *some*? The process bringing to light the SOME BUT NOT ALL interpretation of *some* is bidirectional optimization.

### Bidirectional Optimization: Generating the Implicature

So far, we have seen how two semantic constraints account for children’s production and comprehension of the lexical element of the <*some*, *all*>-scale. Remarkably, according to our analysis, the comprehension of *some* results in ambiguity (see Figure 2B). This ambiguity, however, can disappear in the adult comprehension thanks to a process of bidirectional optimization. In a bidirectional optimization process, the effect of the constraints in production and the effects of the constraints in comprehension are both taken into account (Blutner, 1998, 2000 and subsequent works). Informally, bidirectional optimization can be thought as a perspective-taking mechanism (Van Rij et al., 2010). Let us describe its functioning.

Suppose an opinionated speaker wants to refer to a situation in which three out of five roses are red using an expression on the <*some*, *all*>-scale (and not, say, a cardinal number). In light of the two constraints Strength and FaithAll, the speaker has no choice but to use the form *some*, with its upper-bounded reading (see Figure 1B). Notice that no implicature has been generated here. As we will see in Section “Discussion,” producing *some* to refer to a subset is not equivalent to generating an implicature.

What about the hearer? When hearing *some*, the hearer’s language system is faced with an ambiguity (see Figure 2B): from the hearer’s perspective, given Strength and FaithAll, it is impossible to choose between a complete or a non-complete set. However, *some* can be disambiguated if the hearer considers also the speaker’s perspective.

**FIGURE 3:**
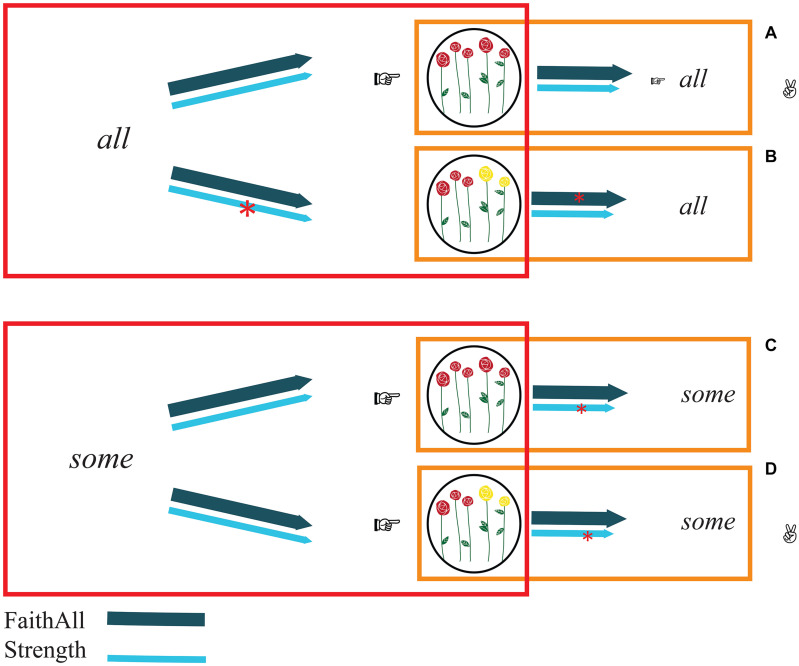
Bidirectional optimization (in comprehension). Panels **(A–D)** represent the four possible form-meaning associations. The process starts with an evaluation of the optimal meanings given the forms *all* and *some* (red boxes) and continues with a second step in which the associations are evaluated according to speakers’ perspective (orange boxes). The first bidirectionally optimal association that can be determined is panel **(A)**: it does not violate any constraint in comprehension (see red boxes) nor in production (see orange boxes). All the other possible associations violate at least one constraint (see red asterisks). However, if hearers also consider the speaker’s perspective, panels **(B,C)** must be excluded. Hence, panel **(D)** emerges as the second optimal association: there is no better meaning than reference to the non-complete set to interpret *some*, and there is not better form than *some* to refer to a non-complete set of elements.

To do so, the hearer needs to consider the effects of the constraints Strength and FaithAll not just from the hearer’s own perspective (comprehension perspective, in which the output is a meaning) but, simultaneously, also from the speaker’s perspective (production perspective, in which the output is a form). So, rather than simply finding the optimal meaning of the form the hearer has heard, the hearer needs to assess whether, in production, that optimal form would have been chosen for that meaning. In other words, the hearer needs to evaluate all *form-meaning associations* on the basis of the constraints (see Figure 3). Concretely, the bidirectional process proceeds as follows. Taking into consideration both hearer’s and speaker’s perspective, a first optimal association, which does not violate any constraint, can be identified: the association between the complete sets and the form *all* (association in Figure 3A). Globally, both from the production perspective and from the comprehension perspective, this association does not violate Strength (“Use the strongest element on the Horn scale”) or FaithAll (“*All* corresponds to complete sets”). For example, in production, uttering “All roses are red” to refer to a complete set of roses does not violate any constraint. Likewise, in comprehension, interpreting “All roses are red” as referring to a complete set of roses does not violate any constraint.

What about the other associations? The other possible associations are the following: *all-*non-complete set (Figure 3B), *some-*complete set (Figure 3C), and *some-*non-complete set (Figure 3D). Crucially, the first two (Figures 3B,C) must immediately be excluded. Specifically, the association *all-*non-complete set (Figure 3B) cannot be considered an optimal association because the form *all* can be better interpreted as referring to the complete set (i.e., association in Figure 3A). Likewise, the s*ome-*complete set (Figure 3C) cannot be considered an optimal association because the complete set can be better referred to using *all* (so, Figure 3A). By exclusion, then, the association between *some* and the non-complete set (Figure 3D) can be established. As a matter of fact, there is no better interpretation for *some* than the non-complete set, and there is no better form than *some* to express the non-compete set.

Due to this process of bidirectional optimization, the hearer has considered all the possible ways in which *some* and *all* can be interpreted (the associations in Figures 3A–D) and can conclude that in uttering *some*, the speaker could have in mind only one of *some*’s meanings. Thus, the hearer is able to associate the word *some* with the upper-bounded reading (Figure 3D). SI generation consists precisely in this disambiguation of *some* on the part of the hearer.

One observation is now in order. That hearers and speakers have different roles in SI generation is undisputed. As Horn (2006) rightly claimed: “Speakers implicate, hearers infer.” Nonetheless, the idea that production and comprehension are distinct processes has not been incorporated in theories of implicatures. Most importantly, it hardly plays a role in any explanations of children’s difficulties. We will now discuss the advantages of our Bi-OT approach, which allows one to consider the production and comprehension processes separately.

## Discussion

The constraints we introduced in Section “Translating Horn Scales in Constraints,” FaithAll and Strength, and their interaction, are at the core of our Bi-OT analysis. In this section, we examine in detail the predictions that arise from our Asymmetry Account, concerning in particular the *some*-implicature asymmetry (see section “When Speakers Are More Logical Than Hearers”), the relationship between ToM and implicature generation (see sections “Scalar Implicature Generation and Theory of Mind” and “When Speakers Become Less Logical and More Pragmatic”), children’s variable performance in comprehension studies (see section “Explaining Children’s Variable Performance in Comprehension Studies”), and children’s interpretation of numerals (see section “Why Children Interpret *n* as EXACTLY *n*”).

### When Speakers Are More Logical Than Hearers

In Bidirectional Optimality Theory, the same set of constraints can affect production and comprehension differently (Smolensky, 1996; Hendriks, 2016). In presenting our Asymmetry Account, in line with Hendriks and Spenader (2006), we maintain that Bi-OT has psychological validity and should not be considered merely in a diachronic perspective (cf. Blutner, 2010, and subsequent works).

Specifically, we argue that as soon as children master the two semantic constraints Strength and FaithAll, they start to produce *all* and *some* in an adult-like manner. Importantly, this means that they are able, in production, to use *some* with its upper-bounded meaning (SOME BUT NOT ALL) from a very early age. However, as we have seen, on the basis of Strength and FaithAll, the form *some* happens to be ambiguous in comprehension. Consequently, in the early stages of language acquisition, the child language system cannot distinguish between the two possible interpretations of the quantifier *some*. In order to acquire the ability to comprehend *some* as adults do, children need to acquire the ability to carry out bidirectional optimization, which can be seen as the formalization in OT of perspective-taking (Hendriks et al., 2010). Only when children optimize bidirectionally they can generate an implicature, interpreting *some* with the upper-bounded meaning.

The first prediction of our analysis, then, is the following: no complex inferential process is needed in order to produce *some* with its upper-bounded meaning. In this, a clear asymmetry emerges. The comprehension of *some* requires perspective-taking (in the form of bidirectional optimization) and is thus more complex than the production of *some*. This can explain the findings of the elicitation task of Foppolo and Guasti (2005), Katsos and Smith’s (2010) production results, as well as corpus data presented by Eiteljoerge et al. (2018). In all these studies, children demonstrated an ability to produce *some* with its upper-bounded reading at an age at which they cannot yet interpret *some* associating it with its upper-bounded reading.

A related important observation is that the adult-like production of the form *some* with its upper-bounded meaning is not equivalent to the production of an implicature. When speakers produce *some* with its upper-bounded meaning, their production of *some* makes *hearers* generate an implicature in order to arrive at the SOME BUT NOT ALL meaning. Nonetheless, speakers do not generate implicatures themselves. As the language acquisition findings of Eiteljoerge et al. (2018) suggest, for an implicature to emerge, speakers do not need to have an *intention* to produce implicatures (to *implicate*). Consequently, *contra* Hirschberg (1985) and Horn (2006), we believe that shifting the focus from speakers’ intentions to hearer’s perspective-taking process can be greatly beneficial in defining and studying implicatures. The different roles of hearers and speakers in implicature generation will be discussed further in Section “When Speakers Become Less Logical and More Pragmatic.” Before doing that, it is worth considering more in detail the relation between the ability to generate implicatures and a particular cognitive ability: ToM.

### Scalar Implicature Generation and ToM

So far, we claimed that the ability to generate implicatures develops with age in parallel with the ability to optimize bidirectionally and that bidirectional optimization can be seen as a kind of perspective-taking mechanism. In light of this, it is worth considering the role played by ToM, broadly defined as the understanding of others’ feelings, desires, intentions, and beliefs (Wellman, 2018).

Evidence suggesting a connection between bidirectional optimization and ToM comes from another production–comprehension asymmetry in child language, namely, the asymmetry observed with object pronouns. Despite the fact that children experience problems with the interpretation of object pronouns until at least the age of 6 (see Hamann, 2011, for an overview), children’s pronoun production is almost adult-like from the age of 4;6 (Spenader et al., 2009; cf. De Villiers et al., 2006). This asymmetry has been accounted for in the framework of Bi-OT by Hendriks and Spenader (2006). These authors claimed that pronoun interpretation, but not pronoun production, requires bidirectional optimization, and it is inextricably linked to ToM. Hendriks and Spenader (2006) prediction found experimental support in the study of Kuijper (2016), who demonstrated the existence of a correlation between pronoun interpretation and ToM skills in children. If, as we claim, also SI generation depends on bidirectional optimization, then we expect to find correlations between children’s ability to generate SIs (in comprehension) and their ToM abilities. It is worth mentioning that, in contrast, we do not expect ToM to be correlated with the adult-like production of *some*, because production does not require complex inferential processes.

Studies on Autism Spectrum Disorders (ASD) can perhaps be useful in verifying whether, as we argue, the ability to generate SIs relies on ToM. Given that ToM deficits are considered core symptoms of ASD (Baron-Cohen et al., 1985; Frith, 2001), individuals with ASD are expected to show difficulties with SI generation. At first sight, the studies of Pijnacker et al. (2009) and Chevallier et al. (2010) fail to support this hypothesis. In both studies, the performance in SI generation of adult or adolescent participants with ASD did not differ from the one of neurotypicals. Nevertheless, two observations are in order. First of all, in these two studies, participants’ ToM levels had not been assessed; hence, it is possible that a correlation between SI generation rate and ToM skills was present at the individual level. Besides, it is conceivable that individuals with ASD adopted a different strategy to generate implicatures, or better, to associate *some* with the SOME BUT NOT ALL meaning, without actually having to perform perspective-taking (cf. Hochstein et al., 2018, on epistemic reasoning in ASD).

Furthermore, some results pointing to a correlation between ToM and SI generation, at least in children, do exist. In Pastor-Cerezuela et al. (2018) the ability to generate different kinds of QBIs was assessed in TD children and children with ASD. The performance of children with ASD was significantly lower than the performance of age-matched and language-matched TD children (see also Surian et al., 1996, on the “deafness” of children with ASD to Gricean Maxims).

Even stronger evidence for the existence of an association between ToM and SIs in language acquisition comes from the recent study of Foppolo et al. (2020). Importantly, this study was the first to systematically assess in monolingual TD children the possible correlations between SI generation, on one hand, and linguistic and cognitive abilities (lexical and morphosyntactic comprehension, IQ, and first-order ToM), on the other. In the group of preschoolers (i.e., before the age of 6, when the ability to generate SIs is still feeble and ToM still developing), first-order ToM abilities (which were found to be independent from lexicon, morphosyntax, and IQ measures) correlated with the ability to generate SIs^2^. Hence, these experimental data speak in favor of an association between ToM and children’s SI generation abilities.

This being said, concerning the relationship between perspective-taking and SIs, we should sound a note of caution. It is surely conceivable that bidirectional optimization as a kind of perspective-taking process could gradually become more automatic, not only in individuals with ASD, but also in the neurotypical adult language system (cf. Blutner’s, 2006 hypothesis of fossilization). This could indeed speed up and hence facilitate adults’ SI generation. As said, it is generally acknowledged that SIs are quite context-independent, precisely because they are rather lexicalized. After all, if “today’s morphotactics is yesterday’s syntax” (Givón, 1971, p. 25), then today’s semantics is yesterday’s pragmatics. Nonetheless, we are inclined to believe that SI generation remains a cognitively costly two-step process (see Hendriks, 2014, for the claim that bidirectional optimization can be conceived as a two-step mechanism). Several studies on adults’ SI generation seem to support this idea (Huang and Snedeker, 2009; Tomlinson et al., 2013; cf. Chemla and Bott, 2014 and van Tiel et al., 2019). Moreover, processing effort is most likely to re-emerge whenever a SI is canceled. This and the related issue of recursive ToM in adults is the focus of the next section.

### When Speakers Become Less Logical and More Pragmatic

We have argued that, for an implicature to emerge, hearers need to take the perspective of the speaker (through bidirectional optimization), whereas speakers can remain “logical.” However, this does not necessarily mean that, in general, speakers do not intend implicatures to be generated. Surely, reasoning recursively about their interlocutor, adult speakers *can* intend an implicature.

Let us take Grice’s (1989) famous example of a philosophy professor who is asked to provide a recommendation letter and, in describing the abilities of a student who is applying for a philosophy job, writes: “Dear Sir, Mr. X’s command of English is excellent, and his attendance at tutorials has been regular. Yours, etc.” (Grice, 1989, p. 33). Obviously, in this case, it is evident that the philosophy professor wants the reader to infer that the pupil is actually not suited for the job. Undoubtedly, then, the philosophy professor intends an implicature to be generated. In writing the letter, the professor is reasoning, in a recursive fashion, about the reader’s reasoning about the professor’s own words. Specifically, the professor wants the reader to recognize that the professor wants the reader to think that the pupil is not suited for the job. In terms of ToM abilities, this corresponds at least to second-order ToM. Second-order ToM is the ability to understand other people’s intentions/beliefs about other people’s intentions/beliefs. Complex as it may be, this kind of recursive mind reading is within the reach of the average adult cognitive capacity. Human adults are talented mind readers, despite the fact that this talent appears to have a limit (see Franke and Degen, 2016, for a discussion of recursive reasoning in reference games). Either way, children develop first-order ToM (the ability to understand intention/beliefs) around the age of 4, whereas second-order ToM skills (the ability to understand intentions/beliefs about intentions/beliefs) require at least two more years to develop (Perner and Wimmer, 1985; Sullivan et al., 1994; Tager-Flusberg and Sullivan, 1994) if not more (cf. Flobbe et al., 2008). So, if second-order ToM is involved in those circumstances in which speakers actually intend an implicature to be generated (such as in the case of Grice’s philosophy professor), we can predict that 5-year old children are not yet able to do so.

Moreover, it is worth observing that if a speaker intends an implicature to be generated, the speaker should also be able to consciously cancel the implicature. An implicature can be canceled by adding an expression that conveys the negation of what can be inferred via an implicature (Hirschberg, 1985; Grice, 1989; Mayol and Castroviejo, 2013, for discussion):

(14) Julia misses some of her friends. *In fact, not just some. She misses all of them.*
(15) Dear Sir, Mr. X’s command of English is excellent, and his attendance at tutorials has been regular. *With this, I do not mean to say he has not great potential as a philosopher. He surely has.*

Canceling an implicature presupposes being able to grasp the difference between the semantic content of the sentence and what can be inferred from the utterance. Thus, as emerges from these examples, implicature cancelation seems impossible without considering the hearer’s inference about the speaker’s utterance. Because of the fact that this probably requires at least second-order ToM skills, we expect 5-year-old children not to be able to cancel implicatures. Future research could systematically explore this interesting issue.

The claim that the ability to cancel can be considered a litmus test for the ability to generate SIs is particularly relevant for the discussion concerning the division of labor between semantics and pragmatics in non-human animals. In thought-provoking work, Schlenker et al. (2013) argued that Campbell’s monkey alarm calls can be analyzed as implicature-like phenomena. In another study, Schlenker et al. (2016a) hypothesized that the system of Putty-nosed monkey alarm sequences could be based on an informativity principle (see also Schlenker et al., 2016b). We believe that such claims are in harmony with the general perspective on SIs outlined in this paper. Adopting a weak scalar term to negate the stronger one does not require complex inferential processes; informativeness alone suffices. Interestingly, Schlenker et al.’s (2016a)’ informativity principle appears directly comparable with our constraint Strength. However, turning back to implicature cancelation, it can be observed that human and monkey’s “implicatures” are likely to differ substantially. Given their ToM skills, we could confidently claim that non-human primates are unlikely to be able to reach a level of perspective-shifting sophistication that would allow them to cancel implicatures (for an overview on ToM in non-human animals, see Penn et al., 2008).

### Explaining Children’s Variable Performance in Comprehension Studies

In this section, we will take a closer look at previous studies on children’s SI generation and discuss children’s performance in light of our Asymmetry Account.

One issue we raised at the beginning of this paper concerns the fact that the experimental manipulation adopted when testing children’s comprehension of *some* has substantial influence on children’s performance on the task. Most remarkably, at the same age at which children fail to generate SIs in Truth Value Judgment Tasks (TVJTs), they perform adult-like in Felicity Judgment Tasks (FJTs) (Foppolo et al., 2012). Our account straightforwardly explains the difference in results between these two tasks, and in particular the reason why SI generation is not necessary in FJTs. Let us start by considering the TVJT. For children who cannot shift their perspective, *some* is ambiguous according to our Bi-OT account and can be taken as referring to complete as well as non-complete sets. Consequently, they accept sentences such as “Some chipmunks are taking a shower” when shown a picture in which all five chipmunks are taking a shower. The non-adult-like overacceptance of this sentence in such a context stems from children’s strict adherence to the constraints Strength and FaithAll. Children’s adult-like performance in FJTs is also in line with our account. In this paradigm, children are presented with a visual scenario representing a complete set and asked which of two utterances better matches the visual scenario. One statement contains the quantifier *some*, the other (the most appropriate) the quantifier *all*. The reason why children perform well in this task is that the choice of form does not require perspective-taking and SI generation. When hearing the form *all*, children immediately associate this form with a complete set, thanks to the constraint FaithAll. On the other hand, when hearing the *some*-sentence, they are faced with an ambiguity. On the reasonable assumption that non-ambiguous forms should be preferred to ambiguous ones, it is natural for children to prefer *all* to *some*. In other words, to refer to complete sets, *all* is a better candidate, because it better predicts the complete set.

Katsos and Bishop’s (2011) results can arguably be explained along similar lines. In this study, children were tested using both a classical binary TVJT and a ternary judgment task. Instead of rejecting or accepting sentences, in this second paradigm, participants have a middle answer option, and can reward a puppet who utters *some-* and *all*-sentences using a huge, big, or small strawberry. Notably, despite accepting underinformative *some*-sentences in the binary TVJT, in the ternary judgment task, children preferentially chose the middle option.

In light of our account, it can be argued that, because the comprehension of the form *some* results in two possible meanings (see Figure 2B), children can grasp the ambiguity of *some* even without considering the form *all*, and without generating the implicature. Because of this, they are in fact expected to choose a medium-sized reward for *some*-sentence in such contexts. Note that, while we predict that children’s SI generation abilities correlate with ToM abilities, we do not predict such a correlation between their performance in ternary judgment task and their ToM abilities. Future research could experimentally test this prediction.

Explaining why Pouscoulous et al.’s (2007) action-based task enhances children’s ability to generate SIs is more challenging. However, we will propose a possible explanation. In Pouscoulous et al. ’s (2007) action-based task, 4- to 7-year-olds were presented with different scenarios. In critical trials, children were shown an ALL-scenario (five open boxes containing a token each) and heard a puppet uttering “I would like some boxes to contain a token.” Children’s task was to act on the scenario to comply with the wish of the puppet. Obviously, removing tokens in this scenario means having generated a SI. Children performed extremely well in this AOT. Quite surprisingly, 68% of 4-year-olds and 73% of 5-year-olds demonstrated the ability to grasp the incongruity between the *some*-statement and the ALL-scenario. At first glance, our Bi-OT explanation cannot account for this result. The comprehension of the form *some*, as we have seen, leads to ambiguity, unless a perspective-taking operation takes place. Hence, children’s choice should be simply to leave the scenario as it is. However, by carefully considering what children are required to do in the task, we could argue that this kind of AOT does not require simply a comprehension process. On the contrary, this task instead appears to trigger a production process. The reason is the following: children know that they have been asked to act on the scenario (leaving it as it is, or removing tokens, or adding tokens). Hence, in order to act, children need to actively focus on, reflect on, and act on the visual context. Thus, quite naturally, the elements and characteristics of the visual scenario become for them concepts or meanings. When presented with the ALL-scenario, children obviously recognize a complete set. From this, they can carry out a simple production process and choose the form *all*. In Bi-OT terms, it could be said that this kind of AOT does not consist simply in evaluating the optimal interpretation for a given form (as happens in a typical comprehension process); rather, this task consists in selecting the interpretation that gives rise to the *given form as the optimal form*. The task, thus, triggers a production-like process. Consequently, children’s performance is enhanced. This is clearly in contrast to what happens in a TVJT, because in this case, children are asked to accept or reject a sentence, and there is nothing in the instructions they receive that can trigger a production process (from a given meaning to a potential form for optimally expressing that meaning).

In general, then, it can be argued that in order to explain children’s good performance in particular comprehension tasks, we should always consider whether the instructions or the manipulation encourage children to take as the input a particular meaning, because this is likely to trigger a production process, or, conversely, a particular form, thus triggering a comprehension process. Perhaps the rather vague expression “task demand,” often used to justify children’s variable performance (e.g., Pouscoulous et al., 2007), reflects exactly this: the more production-like the task is, the less children struggle to generate the SOME BUT NOT ALL meaning.

One last remark, concerning the relationship between our account and other accounts of SI generation is in order here. First, as clearly emerges from Section “Translating Horn Scales in Constraints,” the Asymmetry Account attributes a fundamental role to Horn scales. Children’s knowledge of Horn scales (hence, of lexical alternatives) is seen as a prerequisite for children’s ability to optimize bidirectionally over scalar elements. Therefore, it should be emphasized that our account and lexicalist accounts (Barner et al., 2011; but also Foppolo et al., 2012) are not mutually exclusive, but rather can be integrated in a broader perspective on children’s SIs generation. Secondly, we would like to stress that our account, which focuses on children and shows how children’s difficulties are to be related to their developing cognitive abilities (in particular, ToM) can complement other accounts of implicature generation in adults. In particular, the constraint-based, probabilistic approach proposed by Degen and Tanenhaus (2015) seems highly compatible with OT models (see the constraint-based, stochastic version of OT developed by Boersma, 1998).

### Why Children Interpret *n* as EXACTLY *n*

According to some researchers (e.g., Horn, 1972; Levinson, 1983; cf. Horn, 1992), bare numerals are scalar items whose literal meaning is AT LEAST *n*. The EXACTLY *n* meaning of numerals is derived via the standard process of SI generation, through which scalar elements assume upper-bounded interpretations. The process can be exemplified as follows:

(16) Utterance: The boy caught three crabs.
(17) Non-pronounced alternative: The boy caught four crabs.
(18) Inference: The boy caught exactly three (and not four) crabs.

This neo-Gricean view has been challenged on theoretical grounds by proponents of a seemingly simpler approach to numeral interpretation, according to which numerals are not interpreted as other scalar elements: their primary meaning is EXACTLY *n* (e.g., Geurts, 2006; Breheny, 2008), and the other possible readings, such as the lower-bounded AT LEAST *n*, are secondary and derived in various ways (see Spector, 2013, for a discussion of different approaches).

Importantly, the acquisition literature shows that children’s interpretation of numerals does not pattern with their comprehension of other scalar elements. Papafragou and Musolino (2003), for instance, showed that 5-year-old children, despite not being able to reject underinformative sentences containing the scalar term *some* (“Some of the horses jumped over the fence” when three out of three did), tend to reject underinformative sentences containing a numeral (“Two of the horses jumped over the fence” when three out of three did). This result was replicated in various studies. Hurewitz et al. (2006) tested children aged 3;0 to 4;0 adopting a variation of the classical Picture Selection Task. In line with Papafragou and Musolino’s (2003) findings, children performed at chance in the comprehension of *some*, but attributed the exact meaning to the numeral *two*. Even stronger evidence in this direction came from the study of Huang et al. (2013). Adults and children between the ages of 2;6 and 3;5 were tested using a clever paradigm, the Covered Box Task (see Huang et al., 2013, for details). Adults, but not children, demonstrated the ability to generate and also cancel the *some*-implicature. On the other hand, in interpreting the numeral *two*, both children and adults behaved as if an implicature could not be canceled. Both groups always interpreted *two* as only compatible with EXACTLY TWO. Thus, this study shows that it is not simply the case that children merely learn to draw numeral-inferences before the *some*-implicature, as it could have been hypothesized in light of previous studies. Specifically, Huang et al.’s (2013) results yield support for the claim that there exists a true difference between the interpretation of scalar elements such as *some* and the interpretation of numerals. In essence, unlike the former, the latter does not involve implicature generation.

Our account provides a clear explanation as to *why* numerals do not give rise to SIs. Recall that, in our Bi-OT account, Horn scales are defined as scales characterized by the presence of an apex, a culmination point that represents the maximization of the dimensions denoted by the scale. Every time we use a weaker term of a scale, the negated alternative corresponds to the apex. Clearly, if scales do not have apices, they cannot trigger the generation of SIs. Notably, the scale of numerals is unbounded, that is to say, it is a scale without apex. In fact, by definition, in this scale, there cannot be an upper bound: the set of natural numbers, which adults intuitively conceive as being based on the successor function S(*n*) = *n* + 1, is infinite. Consider again the sentence in (16). The numeral *four* is stronger in terms of logical entailment than *three*. However, so is the numeral *five*, the numeral *six*, and so forth, *ad infinitum*. In our terms, the dimension conveyed by this scale has no maximization point. Hence, given that a single, relevant, strongest alternative cannot be identified (because the alternatives are infinitely many), no single, relevant, strongest alternative can be negated. As a consequence, no comparison between the weaker term and its strongest alternative can take place, and we predict that the interpretation of numerals does not require a process akin to SI generation. In other words, it is because of the very semantics of the apex-less scale of numerals that numerals receive an exact semantics.

One final remark is in order. As an anonymous reviewer rightly points out, in the classical experimental setting adopted to test children’s comprehension of numerals, a context-dependent apex can be identified and hence, in principle, an implicature *can* be generated. To give a concrete example, imagine a visual context featuring a boy catching four crabs. Given this scenario, in order to judge an utterance such as (16), “The boy caught three crabs,” it is possible for participants to proceed as follows. First, they can consider the four crabs that are visually salient in the context and regard them as an *ad hoc* apex (i.e., an apex that is not based on a particular Horn scale, but that is contextually relevant). Secondly, participants can carry out a perspective-taking process (bidirectionalization). Finally, having generated an implicature and inferred THREE (AND NOT FOUR), they can reject the utterance (16).

Albeit possible, such a bidirectionalization process would require cognitive skills that are not fully developed in young children. Besides, as predicted by the Asymmetry Account, numerals already receive an EXACTLY *n* interpretation as their primary meaning. Consequently, carrying out such a complex inferential process would be not only cognitively costly but also unnecessary: the results of the bidirectionalization process would correspond to the primary meaning of the form.

Hence, despite arguing that the interpretation of numerals does not require implicature generation, our account sheds new light on children’s comprehension of numerals. It shows that it is precisely because of the unboundedness of the scale of numerals that implicature generation does not naturally take place. If an implicature did arise, it would be superfluous, and, at least for young children with immature ToM skills, cognitively unfeasible.

## Conclusion

In this paper, we proposed the Asymmetry Account, a novel account of SI generation in the framework of Bi-OT. The Asymmetry Account is able to explain the rather puzzling asymmetry between production and comprehension of SIs that emerges in language acquisition. Furthermore, it allows us to make a number of interesting predictions. A crucial feature of our hypothesis is that ToM plays a fundamental role in children’s comprehension of implicatures, but not in their production. Because of this, children are expected to experience difficulties in comprehension, albeit being able to produce *some* with its upper-bounded meaning from a very young age. Furthermore, our account explains why an extremely variable performance emerges in studies testing children’s implicature generation in comprehension: some tasks do not require perspective-taking, or inadvertently elicit a production-like process and enhance children’s performance. Moreover, our Asymmetry Account demonstrates that SI generation is not necessary for the interpretation of numerals.

## Data Availability Statement

The original contributions presented in the study are included in the article/supplementary material, further inquiries can be directed to the corresponding author/s.

## Author Contributions

IM and PH conceived the research questions, developed the theory, and wrote the first version of the manuscript. SAS and SJMK contributed in commenting on and revising the manuscript. All authors approved the submitted manuscript.

## Conflict of Interest

The authors declare that the research was conducted in the absence of any commercial or financial relationships that could be construed as a potential conflict of interest.

## Footnotes

1 The claim that *ad hoc* implicatures and scalar implicatures belong to the same class of phenomena (e.g., Hirschberg, 1985; Geurts, 2010) has been challenged on experimental grounds. Stiller et al. (2015), for instance, showed that children, as young as 3;5 years of age, can generate *ad hoc* implicatures (see also Horowitz et al., 2018, for similar results, but see Katsos and Bishop, 2011; Schaeken et al., 2018, for conflicting evidence). Given the experimental methods adopted in *ad hoc* implicatures studies, however, we believe that children’s success with *ad hoc* implicatures may be achieved through a strategy that does not require implicature generation. In particular, we speculate that children’ good performance could be linked to (and possibly explained by) contrast inferencing (e.g., a contrast between the most relevant features of two visually presented pictures) rather than implicature generation (see Sullivan et al., 2019, for experimental evidence that contrast/exclusion inferences may play a role children’s success in various implicatures studies). See also footnote 2 for further evidence that *ad hoc* implicatures and scalar implicatures may be different phenomena.

2 *Ad hoc* implicatures, on the other hand, did not correlate with ToM. This brings further support to the hypothesis that children make use of different strategies to generate scalar implicatures and *ad hoc* implicatures (see footnote 1).
